# Neural Responses to Consciously and Unconsciously Perceived Emotional Faces: A Spinal fMRI Study

**DOI:** 10.3390/brainsci8080156

**Published:** 2018-08-17

**Authors:** Alyssia D. Wilson, Tiffany A. Kolesar, Jennifer Kornelsen, Stephen D. Smith

**Affiliations:** 1 Department of Psychology, University of Winnipeg, Winnipeg, MB R3B 2E9, Canada; aprecourt@live.ca (A.D.W.); Jennifer.Kornelsen@umanitoba.ca (J.K.); 2 Department of Physiology and Pathophysiology, University of Manitoba, Winnipeg, MB R3T 2N2, Canada; Tiffany.Kolesar@umanitoba.ca; 3 Department of Radiology, University of Manitoba, Winnipeg, MB R3T 2N2, Canada

**Keywords:** spinal fMRI, emotion, cervical spinal cord, unconscious perception, anger

## Abstract

Emotional stimuli modulate activity in brain areas related to attention, perception, and movement. Similar increases in neural activity have been detected in the spinal cord, suggesting that this understudied component of the central nervous system is an important part of our emotional responses. To date, previous studies of emotion-dependent spinal cord activity have utilized long presentations of complex emotional scenes. The current study differs from this research by (1) examining whether emotional faces will lead to enhanced spinal cord activity and (2) testing whether these stimuli require conscious perception to influence neural responses. Fifteen healthy undergraduate participants completed six spinal functional magnetic resonance imaging (fMRI) runs in which three one-minute blocks of fearful, angry, or neutral faces were interleaved with 40-s rest periods. In half of the runs, the faces were clearly visible while in the other half, the faces were displayed for only 17 ms. Spinal fMRI consisted of half-Fourier acquisition single-shot turbo spin-echo (HASTE) sequences targeting the cervical spinal cord. The results indicated that consciously perceived faces expressing anger elicited significantly more activity than fearful or neutral faces in ventral (motoric) regions of the cervical spinal cord. When stimuli were presented below the threshold of conscious awareness, neutral faces elicited significantly more activity than angry or fearful faces. Together, these data suggest that the emotional modulation of spinal cord activity is most impactful when the stimuli are consciously perceived and imply a potential threat toward the observer.

## 1. Introduction

Emotional responses are sensorimotor in nature and serve to enhance the likelihood that an individual will successfully detect and respond to a specific stimulus [1,2]. The importance of these responses can be seen at the neural level, with emotional stimuli receiving preferential processing in numerous regions of the central nervous system [3]. For example, fear- and threat-related stimuli reliably elicit enhanced levels of activity in extrastriate regions related to perception and attention [4,5] as well as in motoric regions related to the planning and performance of behavioral responses [6,7,8,9]. Numerous transcranial magnetic stimulation (TMS) studies have also shown that emotional stimuli and emotional states increase the excitability of the corticospinal tract, suggesting that emotion primes the motoric system in order to facilitate a response [10,11,12,13,14].

Recent research has demonstrated that this emo-motoric system extends to both the cervical [15,16] and thoracic spinal cord [17,18]. Using fear- and disgust-eliciting images, Smith and Kornelsen [16] demonstrated that the perception of negative stimuli leads to an increase in neuronal activity in ventral regions of spinal cord segments C5–C8. This emotion-dependent activity is similar to the enhanced responses in extrastriate and motoric regions of the brain and likely reflects the nervous system preparing motoric responses to the perceived stimuli. Indeed, the emotional modulation of spinal cord activity appears to be quite specific, with images depicting emotional upper-limb movements having a greater effect on the cervical spinal cord (which innervates the upper limbs) than images depicting emotional lower-limb movements [15]. However, although these studies provide insight into the role of the spinal cord in emotional responses, additional research is necessary to further refine our understanding of this system. Specifically, it is necessary to assess whether emotion-dependent spinal cord activity occurs in response to different types of stimuli and to examine the sensitivity of these modulatory effects.

To date, all of the spinal fMRI studies of emotional responses to visual stimuli have utilized complex emotional scenes such as those found in the International Affective Picture System [19]. However, it is possible that the neural responses to the movement implied in many of these images may interact with the neural responses to the emotions depicted in these stimuli [20]. For example, an image of someone punching someone else may activate motoric regions related to perceived movement (i.e., the punch) as well as cortical and limbic regions related to emotion (i.e., perceiving the anger that led to the punch). In order to reduce the role of implied motion in the current research, static (non-moving) emotional faces will be used.

Although emotional faces do not depict movement, such stimuli are still likely to elicit motoric responses in an observer. The emotion conveyed by faces often influences whether an observer will approach or withdraw from that individual (e.g., [21,22]). Consistent with this view, a recent multi-voxel pattern analysis (MVPA) study demonstrated that emotional faces elicited activity in motion-sensitive brain areas including area V5f and the posterior superior temporal sulcus [23]. This study is complemented by a repetitive TMS experiment which found that stimulating the premotor cortex led to improved face recognition [24]. However, the degree of motoric response elicited by faces likely differs across emotions, with fearful and angry faces being particularly salient [25]. Fearful faces suggest that the individual expressing the emotion has detected something threatening in the environment; the perception of these faces is typically associated with activity in the amygdala [26,27] (for a review, see [28]). Anger, on the other hand, involves a potentially threatening emotion being directed by an individual toward the observer; the perception of angry faces has been linked with activity in the insula and inferior occipital gyrus, with some studies reporting activity in the precentral gyrus [29] (see [30] for a meta-analysis of 105 fMRI studies). Thus, while both fear and anger are salient, negative emotions, the target of the potential threat—the actor or the observer—differs. Whether this difference leads to distinct patterns of spinal cord activity is currently unknown.

An additional uncertainty related to the emotional modulation of spinal cord activity is whether the emotional stimuli must be consciously perceived in order to have an effect. Neuroimaging studies of the brain have found that “unconsciously perceived” stimuli can influence neural activity. Indeed, several studies have reported amygdalar responses to unconsciously perceived fearful faces (e.g., [31,32]; see [28] for a review). There is also evidence that “masked” faces (i.e., faces followed by a pattern mask that limits conscious perception) lead to changes in heart rate [33]; these autonomic changes may, in turn, influence activity in the spinal cord [17,34].

In order to test the effects of emotional faces on cervical spinal cord activity, participants completed six spinal fMRI runs consisting of three one-minute blocks of angry, fearful, or neutral faces interleaved by 40-s rest blocks. In half of the runs, individual stimuli were presented for 1000 ms and were therefore easy to perceive. In the other three runs, the individual stimuli were presented for only 17 ms, well below the threshold for conscious perception [35]. If the spinal cord activity noted in previous research was dependent upon the amygdala, then one would predict that fearful faces would trigger action-preparation responses in the cervical spinal cord (which innervates the upper limbs). However, if the spinal cord responses were a result of a perceived (potential) threat to the observer, then larger responses would be anticipated for fMRI runs involving angry faces.

## 2. Materials and Methods

### 2.1. Participants

Fifteen healthy individuals from the University of Winnipeg (10 female, mean age = 20.7, 5 male, mean age = 20.8) with no history of neurological or psychiatric illness participated in the study. Ethics approval was obtained from the University of Manitoba Bannatyne Human Research Ethics Board (project identification code HS18235 (B2014:077); approval received July 8, 2014) and the University of Winnipeg Human Research Ethics Board (multi-site approval received August 26, 2014). All participants provided written informed consent prior to participation in the study. Participants underwent magnetic resonance safety screening at the Winnipeg Regional Health Authority Satellite MRI clinic prior to scanning to confirm they could be safely scanned. Patients were ineligible if they had irremovable metal in their body, were taking antidepressant or antianxiety medication, or were pregnant. Patients were recruited via an Introductory Psychology course and received $50 CAD compensation. Participants were required to have normal or corrected-to-normal vision.

### 2.2. Stimulus Materials

Emotional and neutral pictures used for the current experiment consisted of 60 images selected from the Karolinska Database of Emotional Faces [36]. Three different facial expressions were selected: (1) neutral, (2) angry, and (3) fearful. The same photographic subjects appeared in each of the three emotion conditions. An equal number of male and female photographic subjects were selected for the stimulus set. The same group of faces was presented in two different conditions: (a) conscious perception or (b) unconscious perception.

The mask was created in MS Paint by using a neutral face from the Karolinska Database of Emotional Faces that was not displayed as a stimulus in the study and applying the mosaic and swirl distortion buttons; this process allowed us to control for color and overall shape while making the stimulus unrecognizable as a face.

### 2.3. Study Design and Procedure

**Figure 1:**
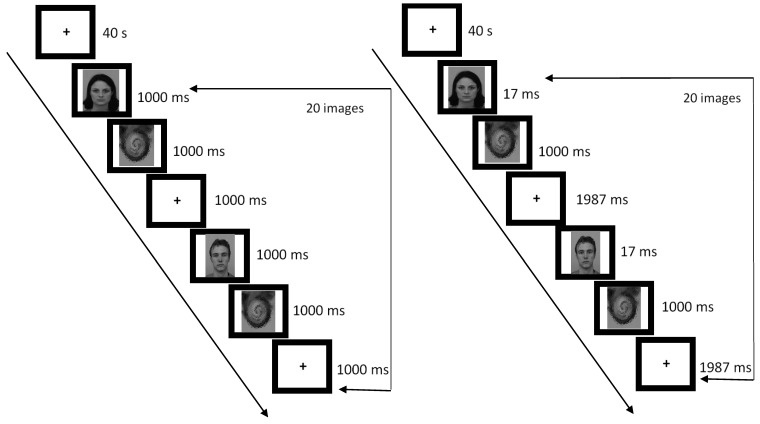
A depiction of sequence of events for Conscious (left) and Unconscious (right) trials. Each trial was 3000 ms in duration; there were 20 trials per one-minute block, and three blocks per fMRI run. A separate fMRI run was performed for each emotion type (Angry, Fearful, and Neutral) at each duration type (Conscious and Unconscious).

Participants completed six different runs during the scanning session; the order of the runs was counterbalanced across participants. Each run was 5 min and 40 s in duration and consisted of three one-minute blocks of stimuli interleaved by 40-s rest blocks featuring a fixation cross. Within each one-minute block, stimuli were presented every three seconds; thus, each block within each fMRI run included 20 unique face images. In the conscious perception trials, an emotional face was shown for 1000 ms, followed by a mask for 1000 ms, and a fixation cross (“+”) for 1000 ms (see Figure 1). In the unconscious perception trials, the face was shown for 17 ms, followed by a mask for 1000 ms, and a fixation cross for 1987 ms.

### 2.4. Data Acquisition and Scanning Parameters

Spinal cord images were acquired on a 3-Tesla whole-body Siemens scanner (TRIO, Siemens Medical Solutions, Erlangen, Germany). Images were acquired using a single-shot fast spin-echo scanning sequence with partial Fourier sampling (HASTE) with the following parameters: TE = 78 ms, TR = 750 ms per slice, FOV = 280 × 210, base resolution = 192 × 192. As this sequence produces images with contrast and signal sufficient for localization of functional activity, no separate structural scan was needed for the overlay of functional data. Nine 2 mm thick contiguous slices were centered rostrocaudally on the C5 vertebra and spanned the cervical spinal cord segments to the corpus callosum. Spatial saturation pulses were applied to eliminate signal from surrounding areas to avoid aliasing, and to reduce motion artifacts arising from regions anterior to the spinal cord.

Cervical spinal cord fMRI data was analyzed with the use of a custom-written Matlab script [37] used in previous studies [15,16,18]. First, images underwent a slice timing correction and were coaligned to correct for bulk motion using a nonlinear 3D adjustment to align each volume to the 3rd volume of the set. Automated normalization was used to match regions of the spatially normalized template to the fMRI images by matching the corpus callosum, midbrain and pons in order to identify the cord in consecutive segments moving from rostral to caudal. After identification of the key structures above, the spinal cord was identified sequentially, based on the previous regions and limited in terms of how much the position and angle of new segments can vary from previous sections. Normalized images (1 mm × 1 mm × 1 mm)—were then compared to the anatomical template as a quality assurance check to confirm proper normalization. Time course data were normalized by applying the spatial normalization parameters to each volume of the series data. Spatial smoothing with a 3D Gaussian filter was applied in the rostro-caudal direction at 2 mm.

Individual level data were analyzed with a General Linear Model (GLM) for each data set to compare the stimuli blocks to the rest periods. Basis functions (modeling the stimulus presentation and convolved with the canonical hemodynamic response function) and noise confounds (based on the first two principal components of the time series data across all voxels) are used to model inherent physiological noise in the data. Analyzed individual results were grouped by condition and different patterns across conditions were compared by running contrasting conditions through a second-level, random-effects analysis. Mean voxel-wise β-values from the individual-level GLMs were averaged and divided by the standard error of the mean to calculate t-statistics between conditions using paired contrasts (*p* = 0.0056 (*p* = 0.05, Bonferroni corrected for the nine contrasts performed), contrast coefficients of −1, 1) [37,38]. Using a spatial extent rationale for balancing rates of Type-I and Type-II errors, contiguous active voxels are considered more likely to be true positives and randomly distributed single voxels are considered more likely to be false positives. Thus, large clusters are likely to represent true activity while small, non-contiguous clusters are more likely to be false positive; for this reason, clusters consisting of one or two voxels have been excluded from results tables. We compared the two negative stimulus conditions with the neutral stimulus condition for both consciously and unconsciously perceived stimuli. We also compared the two negative stimuli runs, anger and fear, against each other in the conscious and unconsciously perceived runs. Significant voxels were manually counted and categorized by type of activity, segment, and physical location.

## 3. Results

Nine contrasts were performed on the data from this study. Each analysis consisted of a Bonferroni-corrected paired contrast (−1, 1). In order to increase clarity, the nine different analyses will be organized into three subsections consisting of (1) a comparison of conscious vs. unconscious perception of the same emotion, (2) a comparison of consciously perceived faces, and (3) a comparison of unconsciously perceived faces.

### 3.1. Conscious vs. Unconscious Perception

The list of active voxels for the three conscious vs. unconscious contrasts are found in Table 1. Clusters containing one or two voxels have been excluded as these are likely to be false positive results. A paired contrast (−1, 1) of the neutral-unconscious and neutral-conscious runs detected 159 significant voxels. Of these, 147 indicated increased activity in the neutral-unconscious condition relative to the neutral-conscious condition and 12 indicated increased activity in neutral-conscious relative to neutral-unconscious condition. Five of the eight clusters indicating greater activity in the neutral-unconscious condition were located in ventral regions, with one large cluster in C5 consisting of 101 voxels. Fewer active voxels were found throughout the cord, except in segment C3. The 12 voxels associated with greater activity in the neutral-conscious condition were all located in the dorsal-right quadrant of the spinal cord segment C1.

A paired contrast (−1, 1) was also performed on the anger-unconscious and anger-conscious runs. This contrast yielded 54 significant voxels, all of which were associated with conscious perception of angry faces. The majority of these voxels appeared in the left ventral regions of the cord, with 27 voxels located in segment C6, and additional activity noted in segments C3 and C5. Additional ventral activity was observed in the medial and right regions of segment C7. No significant voxels were found within the spinal cord for the anger-unconscious condition.

The paired contrast (−1, 1) of the fear-unconscious and fear-conscious runs resulted in surprisingly little activity. In total, only 17 significant voxels were detected. Of these, nine indicated increased activity in the fear-unconscious condition relative to the fear-conscious condition and eight of the voxels indicated increased activity in the fear-conscious condition relative to the fear-unconscious condition. All eight of the voxels linked with the fear-conscious condition were located in the left dorsal or medial spinal cord; five of these voxels appeared in one cluster in C5, while another cluster had two voxels located in C2, and one voxel in C1. The nine voxels associated with greater activity for unconscious perceived fearful faces were located in ventral region of C1, C4, and C6.

Overall, these comparisons of conscious and unconscious perception suggest that consciously perceived anger is more salient than unconsciously perceived anger, and unconsciously perceived neutral faces are more salient than consciously perceived neutral faces. In both of these contrasts, the overwhelming majority of active voxels were located in ventral spinal cord segments associated with action preparedness.

### 3.2. Perceiving Different Emotions: Conscious Perception

**Figure 2:**
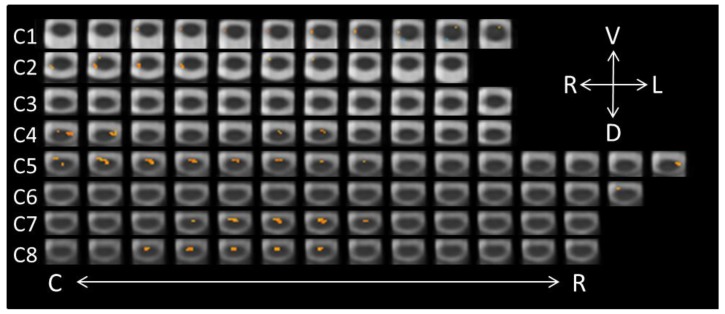
Axial spinal cord slices presenting the distribution of activity produced during the Neutral-Conscious/Anger-Conscious contrast (−1, 1). The images are oriented in radiological convention with dorsal towards the bottom of each frame, left side of the cord on the right of each frame, and are displayed caudal-to-rostral from left to right of each row (α = 0.00556). The abbreviations related to the axial directions (top-right of the figure) are defined as follows: R = right; L = left; V = ventral; D = dorsal. The abbreviations C and R (bottom of the figure) refer to caudal and rostral, respectively.

The list of active voxels for the three contrasts related to the conscious perception of the face-expression stimuli are found in Table 2, again excluding clusters with two or fewer voxels. A paired contrast (−1, 1) was performed on the neutral-conscious and anger-conscious runs. In total, 155 significant voxels were found (see Figure 2), all indicating increased activity in the anger-conscious condition relative to the neutral-conscious condition. The majority of these voxels were located ventrally, with the greatest levels of activity detected in segments C4 (19 voxels), C5 (49 voxels), C7 (32 voxels), and C8 (29 voxels). The dorsal activity associated with conscious-anger was isolated to 15 voxels in the right C2 segment.

A paired contrast (−1, 1) was also performed on the neutral-conscious and fear-conscious runs. The results of this contrast ran counter to our hypotheses. In total, 39 significant voxels were found, all indicating increased activity in the neutral-conscious condition relative to the fear-conscious condition. Although most of the voxels were dorsomedial (C5), two of the three clusters contained ventral voxels; one cluster in C4, and the other small cluster spanning C6/C7.

**Figure 3:**
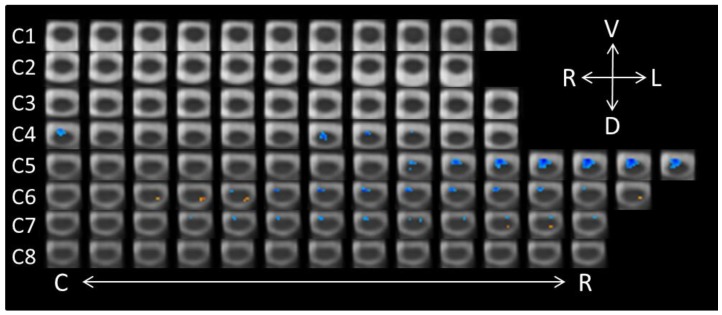
Axial spinal cord slices presenting the distribution of activity produced during the Anger-Conscious/Fear-Conscious contrast (−1, 1). The images are oriented in radiological convention with dorsal towards the bottom of each frame, left side of the cord on the right of each frame, and are displayed caudal-to-rostral from left to right of each row (α = 0.00556). The abbreviations related to the axial directions (top-right of the figure) are defined as follows: R = right; L = left; V = ventral; D = dorsal. The abbreviations C and R (bottom of the figure) refer to caudal and rostral, respectively.

An additional planned paired contrast (−1, 1) was performed in order to test whether the two different negative emotions—anger and fear—elicited different patterns of spinal cord activity (see Figure 3). This analysis demonstrated unequivocally that angry faces lead to a larger motoric response than fearful faces. Indeed, 253 significant voxels were detected, and all in ventral regions of C4–C7 during the conscious perception of angry faces. No voxels in the ventral spinal cord showed larger responses to fearful than to angry faces. Fearful faces did lead to greater activity in the left dorsal region of C6 (nine voxels) and C7 (three voxels).

Taken together, these contrasts provide clear evidence that consciously perceived angry faces lead to large motoric responses in the cervical spinal cord. These data suggest that perceived threat, and not negative emotions in general, lead to preparatory motoric activity.

### 3.3. Perceiving Different Emotions: Unconscious Perception

**Figure 4:**
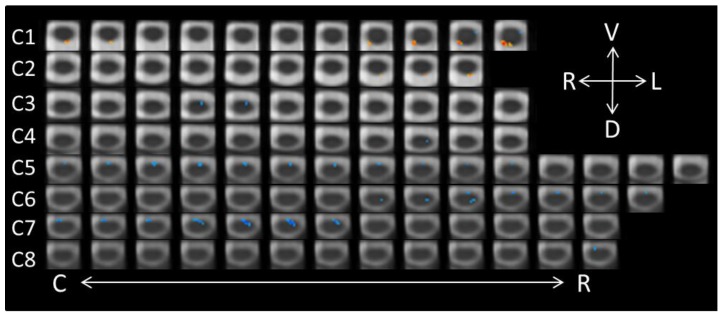
Axial spinal cord slices presenting the distribution of activity produced during the Neutral-unconscious/Anger-unconscious contrast (−1, 1). The images are oriented in radiological convention with dorsal towards the bottom of each frame, left side of the cord on the right of each frame, and are displayed caudal-to-rostral from left to right of each row (α = 0.00556). The abbreviations related to the axial directions (top-right of the figure) are defined as follows: R = right; L = left; V = ventral; D = dorsal. The abbreviations C and R (bottom of the figure) refer to caudal and rostral, respectively.

The list of active voxels for the three contrasts related to the unconscious perception of the face-expression stimuli are found in Table 3, once again excluding clusters containing only one or two voxels. The same three contrasts that were performed for the conscious perception fMRI runs were performed for the unconscious perception runs. First, a paired contrast (−1, 1) was performed on the neutral-unconscious and anger-unconscious runs. In contrast to the results of the conscious perception fMRI runs, the unconscious perception contrast yielded more activity in response to neutral than to negative faces (see Figure 4). In total, 102 significant voxels were found. Indeed, 80 significant voxels were associated with greater activity in the neutral than angry fMRI runs; 22 of the voxels indicated increased activity in the anger-unconscious condition relative to the neutral-unconscious condition. The activity occurring in response to angry faces was isolated to dorsal C1. The unconscious perception of neutral faces, on the other hand, was associated with more activity in ventral, motoric regions. The only dorsal voxels showing greater sensitivity to neutral faces was a group of six voxels in the left dorsal region of C6.

**Figure 5:**
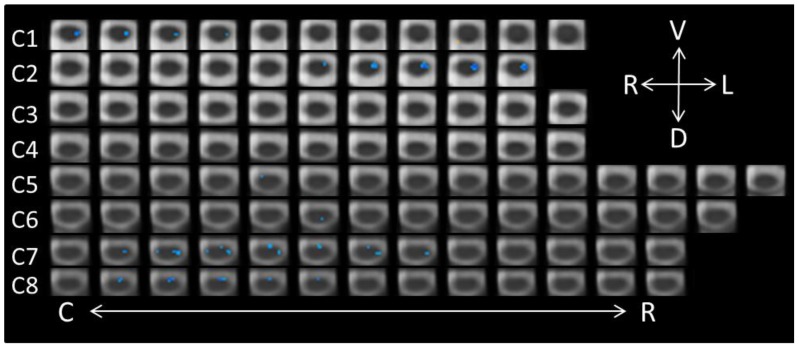
Axial spinal cord slices presenting the distribution of activity produced during the Neutral-unconscious/Fear-unconscious contrast (−1, 1). The images are oriented in radiological convention with dorsal towards the bottom of each frame, left side of the cord on the right of each frame, and are displayed caudal-to-rostral from left to right of each row (α = 0.00556). The abbreviations related to the axial directions (top-right of the figure) are defined as follows: R = right; L = left; V = ventral; D = dorsal. The abbreviations C and R (bottom of the figure) refer to caudal and rostral, respectively.

The paired contrast (−1, 1) of the neutral-unconscious and fear-unconscious runs yielded 86 significant voxels all indicating increased activity in the neutral-unconscious condition relative to the fear-unconscious condition (see Figure 5). Indeed, no significant clusters were observed for the fear-unconscious relative to neutral-unconscious condition. Significant activity was ventral, medial, or ventromedial. The largest cluster spanned segments C1 and C2, with additional activity occurring in C7 and C8.

Finally, a paired contrast (−1, 1) was performed on the anger-unconscious and fear-unconscious runs. This analysis yielded a single significant cluster (four voxels) indicating increased activity in the anger-unconscious condition relative to the fear-unconscious condition. This cluster spanned segments C3 and C4 and was located medially, on the right side.

Taken together, the results of the unconscious perception runs suggest that neutral faces elicit greater motoric responses than fearful or anger faces when they are presented for very brief durations. Potential reasons for this counterintuitive result are discussed below.

## 4. Discussion

The results of the current study suggest that emo-motoric responses are strongest when stimuli are clearly visible and depict a potential threat to the observer. Consciously perceived angry faces elicited robust activity in ventral, motoric regions of the cervical spinal cord, particularly in segments related to movement of the upper limbs and hands. Importantly, this response was not simply a reaction to viewing negative emotions in general. In contrast with consciously perceived fearful faces, angry faces elicited significant activity in 253 ventral voxels, while fearful faces did not lead to greater activity (relative to angry faces) in a single ventral voxel. The primary difference between angry and fearful faces is the target of the potential threat [2,21]. A face expressing fear indicates that the actor is viewing something threatening; a face expressing anger suggests that the actor is directing negative emotion *toward the observer*. This effect is magnified when the gaze of the faces is directed forward [39,40,41], as was the case in the present research. Our results therefore suggest that angry faces are associated with a greater motoric imperative than fearful faces.

The motor responses to threatening faces is consistent with previous research using different methods of measuring neural activity. For example, TMS research has shown that participants produced the greatest motor-evoked potential in response to viewing angry body language compared to fearful or neutral body language [42] (see also [24,25]). Cervical spinal cord responses to anger are also consistent with research using surface electromyography (EMG). Huis in ‘t Veld and colleagues [43] found that the perception of angry body postures lead to increased activity in the biceps, triceps, and deltoid muscles; the perception of fearful body postures elicited activity in the triceps and deltoid muscles, but deactivation in the biceps. In the current study, consciously perceived angry faces elicited much greater activity than fearful or neutral faces in C5 and C7, spinal cord segments that innervate the biceps, triceps, and deltoid muscles, along with several other muscles [44]. This strong spinal cord response suggests that additional surface-based EMG studies are necessary to determine whether any of the other 23 muscles innervated by C5 and C7 show emotion-dependent activity.

Overall, fearful faces elicited surprisingly little activity in the cervical spinal cord. Indeed, consciously perceived fearful faces elicited much less activity than either consciously perceived angry or neutral faces. This result was surprising given that earlier spinal fMRI found pronounced activity in ventral/motoric neurons in segments C2–C5 when participants passively viewed emotionally negative images [16]. A key difference between the results is the stimuli used. Whereas the current result involved the presentation of emotional faces, Smith and Kornelsen [16] used pictorial stimuli from the IAPS image database [19]; it is possible that the movement implied by the complex emotional scenes led to the motoric responses to these stimuli (see [20] for a demonstration of this issue in a brain fMRI study).

It is also possible that the different brain areas involved with perceiving emotional scenes and emotional faces [45,46] could lead to different levels of spinal cord modulation. Given that the amygdala plays a critical role in the perception of fearful faces [26,27] and also shows increased functional connectivity with premotor structures during emotional responses [47], we anticipated that faces expressing fear would be associated with large spinal cord responses. The fact that the opposite occurred suggests that either the amygdala does not modulate spinal cord activity in general, or that these amygdalar outputs are inhibited—potentially by brainstem nuclei [48]—when the emotion does not involve an immediate threat to the individual. Future research involving fMRI scans of the brain and the spinal cord in the same individuals would be necessary to test these competing hypotheses.

Weak responses to fear-related stimuli were also found in the analysis of the unconscious perception fMRI runs. This result was surprising given that a meta-analysis of amygdalar responses noted that the right amygdala was particularly sensitive to fearful stimuli presented below the threshold for conscious awareness [28]. The small effects might be related to the blocked design used in the current study; indeed, the face stimuli were only displayed for a small fraction of each presentation block. Although an event-related design would have allowed us to better isolate the effects of the masked stimuli—and, indeed, to determine if participants’ responses varied as a function of their ratings of each face—the TR in the present study precludes the use of an event-related design. However, the fact that neutral faces presented for 17 ms elicited a robust response compared to angry and fearful faces suggests that despite these limitations, it was possible to detect differences between the experimental conditions.

An additional potential explanation for the relatively weak responses to the unconscious-perception stimuli was that the stimuli were presented too quickly to be perceived, even at an unconscious level. A recent examination of emotional priming effects does suggest that presentation durations can influence the degree to which emotions influence visual perception [49]. Not surprisingly, this research suggests that the ability to accurately encode emotional information from a stimulus is best at durations longer than 200 ms. Importantly, these researchers also noted that when participants reported perceiving a “brief glimpse” of the stimuli, the ability to interpret the displayed emotion was well above chance; the optimal duration for this form of perception was 42 ms. The current research utilized a presentation duration of 17 ms (followed by a pattern mask) in order to ensure that participants did not experience any “fringe consciousness” of the stimuli; this duration was similar to that used in earlier behavioral studies of priming [50,51]. However, it would be informative to perform an additional study in which multiple exposure durations were used (e.g., 17, 34, 51, 68, and 200 ms) in order to identify the temporal point at which the emo-motoric response—at least for angry faces—is initiated. Such a study would also clarify whether these responses are binary (on/off) or vary in intensity as a function of the clarity of the threatening stimulus.

An additional question raised by the current research relates to the large response elicited by unconsciously perceived neutral faces relative to angry and fearful faces. There are two, perhaps complementary, explanations for these effects. First, it is possible that the neutral faces appeared somewhat threatening at rapid presentation durations. There is some evidence in the face perception literature suggesting that the ambiguity of neutral faces does make them appear mildly negative to some individuals [52,53]. This effect could lead to an emotional—and motoric—response in the observer, as seen in the current study. A second possibility is that at very rapid presentation durations, the perception of diagnostic features of emotional faces—for example, the wide eyes in fear and the eyebrows and forehead in anger [54]—may have interfered with the perception of these faces as holistic structures (see [55], for an example of similar effects in a different experimental paradigm). As a result, the rapidly presented neutral faces may have appeared more face-like than the angry and fearful faces. This effect would likely disappear at slightly longer exposure durations, again highlighting the need to explore the emo-motoric responses to faces using a number of different presentation times.

It is important to note that the current research is only an initial step in our attempt to understand how consciously and unconsciously perceived faces influence spinal cord activity. Indeed, this research is not without its limitation. Paramount among these is its reliance on a blocked design used in the current study, the shortcomings of which were noted above. Our investigations would also have been improved with the acquisition of physiological data such as skin conductance responses or measures of cardiovascular changes [56]. Future studies could also require participants to make some form of discriminatory response in order to maintain participants’ continuous attention. These responses could also be combined with an experimental manipulation such as correct vs. incorrect labeling of the emotions, or with participant-generated self-reports of their physiological responses to each stimulus. Doing so would allow us to apply spinal fMRI results to larger theories of emotional experiences, such as the constructionist theory of emotion [57]. These improvements, when combined with variable exposure durations as discussed above, would help address some of the questions arising from this initial investigation of spinal cord responses to consciously and unconsciously perceived emotional facial expressions.

## 5. Conclusions

In conclusion, the current results provide an interesting first step in our understanding of spinal cord responses to facial expressions of emotions. Future studies using fMRI of the brain and spinal cord, more varied presentation durations, and perhaps dynamic facial stimuli [58], would help delineate the situations in which emotional facial stimuli modulate activity in the cervical spinal cord.

## Figures and Tables

**Table 1:** Location of spinal cord activity in the contrasts of consciously and unconsciously perceived faces for each emotion. Results are presented at *p* < 0.0056; results containing 1 or 2 voxels have been excluded as they are likely false positives.

Spinal Cord Segment	Side	Dorsal/Ventral	Voxels
Neutral Conscious > Neutral Unconscious			
C1	Right	Dorsal	12
Neutral Unconscious > Neutral Conscious			
C1	Right	Medial	14
C2	Right	Dorsal	3
	Right	Ventral	4
C4/C5	Left	Ventromedial	8
C5/C6	Right/Medial	Ventral	101
C7	Left	Ventral	3
C8	Right	Dorsal	4
	Medial/Right	Ventral	10
Anger Conscious > Anger Unconscious			
C3	Left	Ventral	7
C5	Left	Medial	5
C6	Left	Ventral	27
C7	Medial/Right	Ventral	15
Anger Unconscious > Anger Conscious			
No active voxels			
Fear Conscious > Fear Unconscious			
C1/C2	Left	Medial	3
C6	Left	Dorsal	5
Fear Unconscious > Fear Conscious			
C1	Medial	Ventral	3
C4	Right	Ventral	3
C6	Right	Ventral	3

**Table 2:** Location of spinal cord activity in the contrasts for the conscious perception of neutral, angry, and fearful faces. Results are presented at *p* < 0.0056; results containing 1 or 2 voxels have been excluded as they are likely false positives.

Spinal Cord Segment	Side	Dorsal/Ventral	Voxels
Anger > Neutral			
C1	Right	Medial	9
C2	Right	Dorsomedial	15
C4	Left/Medial	Ventral	5
C4/C5	Left	Medial	19
C5/C6	Right/Medial/Left	Ventromedial	46
C7	Left/Medial/Right	Ventral	32
C8	Medial/Right	Ventral	29
Neutral > Anger			
No active voxels			
Fear > Neutral			
No active voxels			
Neutral > Fear			
C4	Right	Ventromedial	7
C5	Right	Dorsomedial	27
C6/C7	Left	Ventral	5
Anger > Fear			
C4	Medial	Ventromedial	18
C5/C4	Right	Ventral	161
C6	Right	Ventral	46
C7	Right	Ventral	18
	Left	Ventral	10
Fear > Anger			
C6	Left	Dorsal	9
C7	Left	Dorsal	3

**Table 3:** Location of spinal cord activity in the contrasts for the unconscious perception of neutral, angry, and fearful faces. Results are presented at *p* < 0.0056; results containing 1 or 2 voxels have been excluded as they are likely false positives.

Spinal Cord Segment	Side	Dorsal/Ventral	Voxels
Anger > Neutral			
C1	Right	Dorsal	19
	Medial	Dorsal	3
Neutral > Anger			
C3	Left	Ventral	4
C5/C6	Medial	Ventral	31
C6	Left	Dorsomedial	6
C7/C8	Left/Medial/Right	Ventral	39
Fear > Neutral			
No active voxels			
Neutral > Fear			
C1/C2	Left	Ventromedial	45
C7	Left	Medial	5
C7	Medial	Ventral	9
	Left	Medial	13
C8	Medial/Left	Ventral	14
Anger > Fear			
C3/C4	Right	Medial	4
Fear > Anger			
No active voxels			
